# Assessment of Inter-Expert Variability and of an Automated Segmentation Method of 40 and 60 MHz IVUS Images of Coronary Arteries

**DOI:** 10.1371/journal.pone.0168332

**Published:** 2017-01-20

**Authors:** François Destrempes, Marie-Hélène Roy Cardinal, Yoshifumi Saijo, Gérard Finet, Jean-Claude Tardif, Guy Cloutier

**Affiliations:** 1 Laboratory of Biorheology and Medical Ultrasonics, University of Montreal Hospital Research Center (CRCHUM), Montreal, Québec, Canada; 2 Department of Biomedical Engineering and Cardiology, Tohoku University, Sendai, Japan; 3 Department of Hemodynamics and Interventional Cardiology, Hospices Civils de Lyon and Claude-Bernard University Lyon 1, INSERM Unit 886, Lyon, France; 4 Department of Cardiology and Research Center, Montreal Heart Institute, Montreal, Québec, Canada; 5 Department of Radiology, Radio-Oncology and Nuclear Medicine, and Institute of Biomedical Engineering, University of Montreal, Montreal, Québec, Canada; Shenzhen institutes of advanced technology, CHINA

## Abstract

The objectives were to compare the performance of a segmentation algorithm, based on the minimization of an uncertainty function, to delineate contours of external elastic membrane and lumen of human coronary arteries imaged with 40 and 60 MHz IVUS, and to use values of this function to delineate portions of contours with highest uncertainty. For 8 patients, 40 and 60 MHz IVUS coronary data acquired pre- and post-interventions were used, for a total of 68,516 images. Manual segmentations of contours (on 2312 images) performed by experts at three core laboratories were the gold-standards. Inter-expert variability was highest on contour points with largest values of the uncertainty function (*p* < 0.001). Inter-expert variability was lower at 60 than 40 MHz for external elastic membrane (*p* = 0.013) and lumen (*p* = 0.024). Average differences in plaque (and atheroma) burden between algorithmic contours and experts’ contours were within inter-expert variability (*p* < 0.001).

## Introduction

Acute coronary syndrome is associated with a high risk of recurrent coronary events and is a leading cause of morbidity and mortality [1]. One of the main causes of acute coronary syndromes has been identified as atherosclerotic plaque rupture. It has been shown that one of the main precursors of plaque rupture is a thin fibrous cap (< 65 μm) and a large necrotic core, forming a thin-cap fibroatheroma [1].

Intravascular ultrasound (IVUS) imaging at 40 MHz has been routinely used to assess coronary artery morphology and guide coronary interventions. Based on IVUS imaging, various area-based vessel morphological measures have been recommended [2, 3]. In this clinical context, it is desirable to have an algorithm for segmenting (*i*.*e*., delineating) external elastic membrane (EEM) and lumen contours in IVUS images as a pre-processing step of further image analysis.

Today, IVUS at frequencies ranging from 20 to 40 MHz provides 200 to 70 μm axial resolution, 400 to 200 μm lateral resolution, and 10 to 5 mm penetration, respectively [4, 5]. In view of the dimensions of thin-cap fibroatheromas, it is desirable to improve image resolution, while keeping a sufficient penetration depth. Increasing the frequency, *e*.*g*. to 60 MHz or higher, is expected to improve axial and lateral resolutions at the expense of a decrease in penetration. However, considering that 60 MHz IVUS allows a 2.5 mm penetration depth [6], and that luminal and vessel radii of normal coronary arteries are within an average range of 0.85–2.1 mm [7] and 1.65–2.55 mm [8], respectively, it follows that 60 MHz IVUS imaging of the entire coronary artery wall may be feasible. In fact, it was shown in the previous study [6] that 60 MHz IVUS had an accuracy in diameter estimation comparable or superior to optical coherence tomography in phantoms of diameters larger than 3.04 mm.

Nonetheless, there is a foreseeable drawback that higher frequency ultrasound results in stronger attenuation in the blood and vascular tissues [5]. Moreover, IVUS at 60 MHz is expected to present an image texture distinct from IVUS at 40 MHz. Thus, the present study investigated the performance of an automated segmentation algorithm, based on the Fast Marching Method (FMM) [9], previously adapted to IVUS at 20 MHz [10] and at 40 MHz [11]. Due to the difference in speckle texture at 60 MHz, the previously published segmentation algorithm was adapted. Meanwhile, since manually traced contours by three independent core laboratories were used as gold-standards, difference in inter-expert variability between 40 and 60 MHz data could be assessed in the present study. Specifically, the objectives of this study were: 1) to investigate a relation between inter-expert variability, on one hand, and contour uncertainty as defined with the proposed segmentation model, on the other hand, at both 40 and 60 MHz; 2) to compare inter-expert variability between 40 and 60 MHz data; 3) to compare the algorithmic to expert discrepancy with inter-expert variability at both 40 and 60 MHz.

## Materials and Methods

### Patients

Twenty-three subjects were recruited (14 men, 9 women) for a study sponsored by Silicon Valley Medical Instruments Inc., Fremont, CA, USA (that study was not conducted at neither of the co-authors’ facilities). The mean age of participants was 60 years (range: 41–80 yo). Among these individuals, matched pre- and post-intervention pullbacks acquired at both 40 and 60 MHz from eight patients were available, for a total of 32 loops. This study was conducted in accordance with the principles of the Declaration of Helsinki and with all applicable regulatory requirements. All participants signed informed consents prior to inclusion. A national ethics committee of the Ministry of Health approved the investigational plan, including the informed consent document, prior to study initiation. Subjects with a history of coronary artery disease were screened for inclusion into this trial. The inclusion criteria at the time of eligibility, and the subject exclusion criteria are listed in Table 1.

**Table 1:** List of inclusion and exclusion criteria.

	**Inclusion criteria at the time of eligibility**
**1**	male or female patients aged ≥18 to ≤80 years
**2**	patients with reference vessels > 2.0 mm in diameter
**3**	patients who were candidate for elective coronary diagnostic and therapeutic procedures
**4**	patients able and willing to comply with all study requirements
	**Subject exclusion criteria**
**1**	female patients that were pregnant or nursing
**2**	evidence of coronary thrombus
**3**	angiographic evidence of total occlusion prior to device insertion
**4**	angiographic evidence of dissection not treated with stent prior to device insertion
**5**	documented history of ejection fraction less than 30%
**6**	left main disease greater than 40% for left coronary target vessels
**7**	angiographic evidence of extreme target vessel tortuosity
**8**	patients who had documented history of significant coronary artery spasm (treated for coronary artery spasm)
**9**	patients who were not able to undergo coronary intervention or coronary artery bypass grafting
**10**	co-morbid conditions that placed the subject at an unacceptable surgical risk (*e*.*g*. severe chronic obstructive pulmonary disease, hepatic failure, immunosuppressive abnormalities, and hematological abnormalities)
**11**	patients suffering from renal insufficiency (creatinine >2.5 mg/dL) or patients with chronic renal failure undergoing dialysis
**12**	patients being treated for active systemic infection, bacteremia or sepsis
**13**	patients with an history of bleeding diathesis or coagulopathy
**14**	any clinical evidence that the investigator feels would place the patient at increased risk with the use of the IVUS device
**15**	prior participation or ongoing in any study involving an investigational drug or device, which may impact the endpoints of this study

### IVUS acquisitions

Acquisitions were performed with a Silicon Valley Medical Instruments High-Definition Intravascular System and proprietary Kodama^™^ catheters (ACIST Medical Systems, Eden Prairie, MN, USA). The same echograph and the same broadband transducer were used for acquisitions at 40 and 60 MHz; only the excitation frequency was changed. As reported in [12], an axial resolution of less than 100 μm can be achieved at 60 MHz. Pullbacks were acquired at a speed of 0.5 mm/sec at a rate of thirty images per second in the right coronary artery (*n* = 2), left anterior descending artery (*n* = 2) or circumflex artery (*n* = 4). All data were anonymized prior to be sent to the Laboratory of Biorheology and Medical Ultrasonics of the University of Montreal Hospital Research Center for image and statistical analyses.

### Experts’ gold-standards

A subset of images from each available pullback (one image every 30 frames or 0.5 mm incremental step) was manually blindly segmented by experimented technicians of the IVUS Core Laboratory at the Montreal Heart Institute (Dr Tardif’s Laboratory), by Dr. Finet at the Cardiovascular Hospital Louis Pradel of Lyon, and by Dr. Saijo at Tohoku University of Sendai. Overall, 2312 images were selected for the validation (68,516 / 30 images) of the 32 loops. Instruction was given to the experts not to trace contours if ever the visual perception of the image (and of the video sequence) did not allow the manual segmentation of either contour (lumen-intima or EEM-adventitia). The manual segmentation software used by each core laboratory was implemented on Matlab (version 7.10 (R2010), the MathWorks, Natick, MA, USA).

### IVUS analysis

The algorithmic IVUS contour detection was addressed with a multiple interface fast-marching segmentation model [10]. The external elastic membrane of the vessel wall (EEM or outer layer of the media) and the lumen boundaries were provided as segmentation outputs. The vessel wall boundaries were modeled as layered contours that propagate under a speed function in the Fast Marching Method (FMM) framework [9]. The multiple interfaces were propagated in the IVUS series of images after having been initially positioned using approximate manual points selected on 2 longitudinal views (L-views) of the 3D volume [11]. The procedure for initializing contours in L-views used dynamic programming to ease user interaction; *i*.*e*., contours were viewed as minimal cost paths and were computed with “Floyd’s algorithm” (see Section 8.2 in [13]). Details on the adapted segmentation algorithm and on the initialization process are given in Appendix A and B, respectively.

The implemented segmentation allowed the possibility of displaying portions of lumen and EEM contours that could be less reliable based on the FMM speed function. Indeed, in the framework of the FMM algorithm, the minimal speed function at convergence can be interpreted as pointwise contour uncertainty (PCU). This latter measure is based on an edge-detector filter and a textural gradient, each one adapted to the type of contour (*i*.*e*., either EEM or lumen).

Local inter-expert variability (local IEV) for each radial scanline from the center of a cross-sectional image was defined as the maximum pairwise distance between the three corresponding points on the manually delineated contours by experts. Since there were 3 expert core laboratories, there were 3 pairwise differences for a given scanline, or 1 if only two experts could trace the contour. The local IEV corresponding to those points of the segmented contours that presented the highest 20% PCU measure was compared with the remaining values (80% lowest PCU) to evaluate if uncertain portions of boundaries yielded higher variability. Quintiles were determined separately for lumen and EEM contour points. Based on these quintiles, one obtains two cut-off values (one for the lumen and one for the EEM contour) for displaying high PCU.

For the purpose of comparing inter-experts variability (IEV) between the 40 and 60 MHz datasets, the distances between all pairs of traced experts' contours on a given cross-sectional image were measured. The maximum of these pairwise distances was considered as the IEV on the given cross-sectional image. This metric is identified as IEV to avoid confusion with the local IEV defined earlier. Note that the notion of distance between two contours refers here to the so-called "point-to-point mean distance" defined in [14].

The average distance between algorithmic and experts’ manually traced contours was used as a measure of algorithmic to expert discrepancy (AED). The average distances were compared with IEV measures on each cross-sectional image for both 40 and 60 MHz data. Only frames with at least two traced experts’ contours were used for the computation of AED, since the IEV measurement requires at least two experts’ contours.

Finally, four clinically relevant measurements were considered: 1) EEM cross-sectional area (CSA); 2) lumen CSA; 3) plaque plus media (or atheroma) CSA; and 4) the plaque (or atheroma) burden [2, 3]. For convenience, we also use the terminology of plaque CSA and plaque burden. Average differences (in absolute value) of these measurements between algorithmic and experts’ contours were compared with the inter-expert variability in the same measurements. The latter notion was defined as the maximal pairwise difference in measurement between the 3 experts.

### Statistics

Wilcoxon rank sum tests were performed on local inter-expert variability between all points with 20% highest FMM contour uncertainty, and the points with 80% lowest FMM contour uncertainty. The IEV measures on 40 MHz data were compared with the ones on 60 MHz data by performing Kruskal-Wallis one-way ANOVA tests on ranks. For each of the two frequencies (40 and 60 MHz), the inter-expert variability in each of the four clinical measurements was related to the average EEM CSA with a linear regression. For each of the two frequencies, the average distances between algorithmic and experts’ contours were compared with IEV measures using Wilcoxon rank sum tests. For each frequency, the average differences in each of the four clinical measurements between algorithmic and experts’ contours were compared with corresponding inter-expert variability using Wilcoxon rank sum tests. All statistical tests were performed with SigmaStat software (version 12.0, Systat Software, San Jose, CA, USA).

## Results

### Experts’ gold-standards

**Fig 1:**
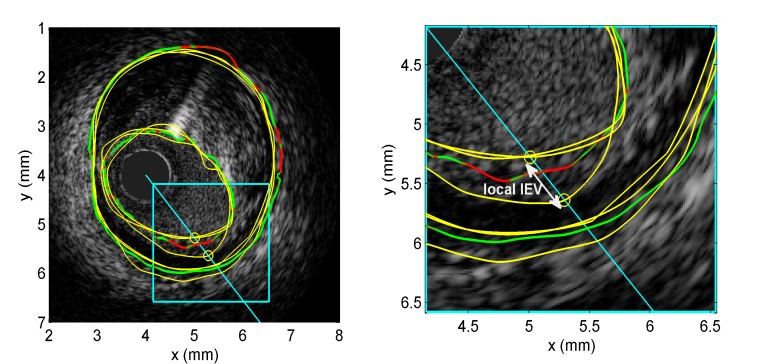
Local inter-expert variability and contour uncertainty. Example of 3 expert external elastic membrane (EEM) and lumen contours and corresponding lumen local inter-expert variability (local IEV) on a given scanline, together with the algorithmic contour with uncertain contour points displayed in red. On the left panel, the 3 experts’ contours (in yellow) and the corresponding algorithmic contours (in green) are displayed for both the EEM and lumen. The points of the algorithmic contour that have high uncertainty are displayed in red. A scanline is shown in cyan, together with the 3 points (circles in yellow) of experts’ contours intersecting the scanline (2 of these points almost coincide in this example). The right panel is a zoomed portion of the left image that lies within the cyan rectangle. The LIEV is the maximal pairwise distance between the 3 points. This measure can be computed for each scanline (corresponding to an angular position within the two dimensional IVUS image).

Out of 2312 frames to be manually segmented, 1155 and 1157 corresponded to 40 MHz and 60 MHZ data, respectively. At 40 MHz, there were 101 (8.7%) or 100 (8.7%) frames without at least two traced experts’ EEM or lumen contours, respectively. At 60 MHz, there were 227 (19.6%) or 216 (18.7%) frames without at least two traced experts’ EEM or lumen contours, respectively. Fig 1 shows an example of 3 manually traced EEM and lumen contours by experts (in yellow). The notion of local inter-expert variability on a scanline is illustrated on this figure; namely, it is the maximal pairwise distance between the 3 contour points lying on the scanline. The corresponding algorithmic contour is also shown on this figure (in green). The points on the algorithmic contour that belong to the 20% highest contour uncertainty are displayed in red instead of green.

### IVUS analysis

**Fig 2:**
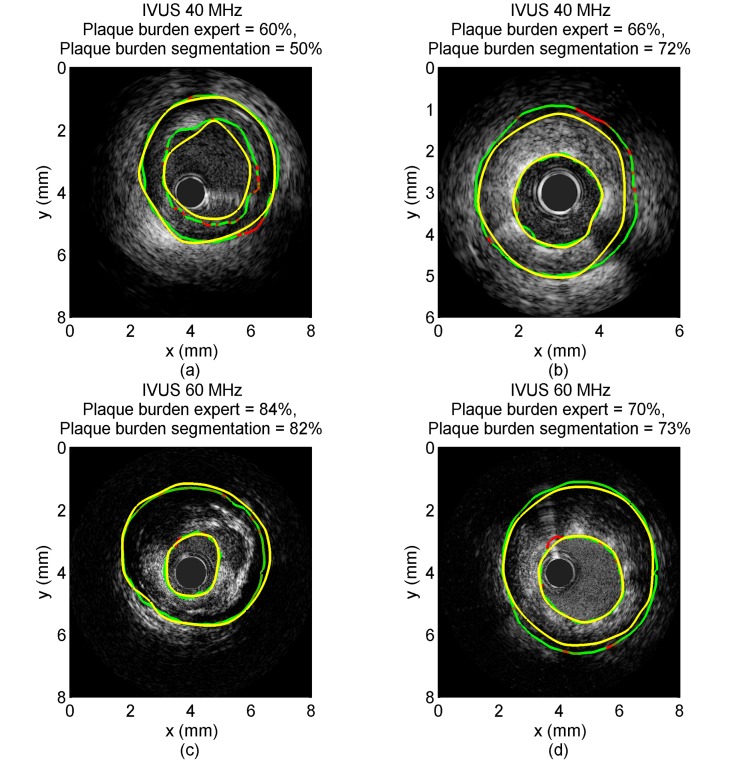
Example of IVUS images with gold standard and segmentation contours. Each panel corresponds to a different patient. Panels a and b display IVUS images acquired with a 40 MHz probe; the image in panel a was acquired pre-intervention, whereas panel b shows a post-intervention acquisition. Panels c and d display IVUS images acquired with a 60 MHz probe; the image in panel c was acquired pre-intervention whereas panel d shows a post-intervention acquisition. The external elastic membrane (EEM) and lumen contours computed with the fast-marching method (FMM) algorithm are superimposed on the images (green full curves with uncertainties in red) together with the EEM and lumen contours manually drawn by an expert (yellow full curves). The plaque burden computed from the segmentation contours and from the expert’s curves is displayed in each panel. The plaque in panel c contains a lipid pool visible from 1 to 4 o’clock. Both post-intervention images (panels b and d) were acquired in a stented vessel segment.

Fig 2 displays examples of algorithmic EEM and lumen contours together with manually traced contours from various experts. On each segmented boundary, the points where the FMM contour uncertainty (*i*.*e*., the FMM speed function) was in the highest quintile are indicated in red.

### Statistics

Local inter-expert variability was significantly higher on the points with 20% highest FMM contour uncertainty than on the remaining points (*p* < 0.001 for both EEM and lumen contours); see Tables 2 and 3 for 40 MHz and 60 MHz datasets, respectively. For EEM contours, the local IEV was 1.6 times greater on average at 40 MHz on points with 20% highest PCU versus remaining contour points (0.203 mm versus 0.129 mm), whereas it was 1.5 times greater at 60 MHz (0.193 mm versus 0.125 mm). For lumen contours, the local IEV was amplified 1.2 times at 40 MHz and 1.1 times at 60 MHz on uncertain contour points determined by the automated algorithm.

**Table 2:** Comparison of local inter-expert variability with pointwise contour uncertainty at 40 MHz.

40 MHz data	EEM local IEV (mm)	Lumen local IEV (mm)
Points with 20% highest PCU	0.203 ± 0.216 (*N* = 115,814)	0.184 ± 0.171 *(N* = 116,326)
Points with 80% lowest PCU	0.129 ± 0.117 (*N* = 463,258)	0.159 ± 0.147 (*N* = 465,306)
*P*-values	*p < 0*.*001*	*p < 0*.*001*

Wilcoxon rank sum tests for comparison of external elastic membrane (EEM) and lumen local inter-expert variability (local IEV) between points with highest 20% pointwise contour uncertainty (PCU) and the remaining boundary points (40 MHz dataset). For both EEM and lumen contours, the local IEV mean value and the standard-deviation (SD) are indicated. The variable *N* represents the number of contour points.

**Table 3:** Comparison of local inter-expert variability with pointwise contour uncertainty at 60 MHz.

60 MHz data	EEM local IEV (mm)	Lumen local IEV (mm)
Points with 20% highest PCU	0.193 ± 0.195 (*N* = 110,899)	0.167 ± 0.145 *(N* = 116,634)
Points with 80% lowest PCU	0.125 ± 0.109 (*N* = 443,597)	0.152 ± 0.138 (*N* = 466,534)
*P*-values	*p < 0*.*001*	*p < 0*.*001*

Wilcoxon rank sum tests for comparison of external elastic membrane (EEM) and lumen local inter-experts variability (local IEV) between points with highest 20% pointwise contour uncertainty (PCU) and the remaining boundary points (60 MHz dataset). For both EEM and lumen contours, the local IEV mean value and the standard-deviation (SD) are indicated. The variable *N* represents the number of contour points.

The inter-expert variability on 60 MHz data was significantly lower than on 40 MHz data for both EEM (*p* = 0.013) and lumen (*p* = 0.024) contours; see Table 4. As an additional test, inter-expert variability in EEM CSA was also found lower (*p* = 0.001) on 60 MHz data compared to 40 MHz data. However, there were no differences in inter-expert variability for the lumen (*p* = 0.091) and plaque (*p* = 0.458) CSAs, or for the plaque burden (*p* = 0.688) between 40 and 60 MHz data. When restricting this analysis on cross-sectional images of 40 and 60 MHz pullbacks corresponding to manually selected matched intervals on which plaque burdens were greater or equal to 80% of its maximal value, the IEV for EEM contours was still significantly lower on 60 MHz data than on 40 MHz data (*p* = 0.049), but there was no significant difference in IEV for lumen contours (*p* = 0.138). Matching of these intervals, which was based on a display of the plaque burden curves along pairs of 40 and 60 MHz pullbacks, could be performed on 14/16 of these pairs of loops. Plaque burden curves were computed from the algorithmic contours. Inter-expert variability in EEM CSA was also found lower (*p* = 0.036) on 60 MHz data compared to 40 MHz data. There were no differences in inter-expert variability for the lumen (*p* = 0.711) and plaque (*p* = 0.074) CSAs, and for the plaque burden (*p* = 0.121).

**Table 4:** Comparison of inter-expert variability between 40 and 60 MHz data.

	EEM IEV (mm)	Lumen IEV (mm)
40 MHz data	0.124 ± 0.077 (*N* = 1155, 101 missing)	0.136 ± 0.080 (*N* = 1155, 100 missing)
60 MHz data	0.120 ± 0.079 (*N* = 1157, 227 missing)	0.129 ± 0.081 (*N* = 1157, 216 missing)
*P*-values	*p* = 0.013	*p* = 0.024

Kruskal-Wallis one-way ANOVA tests on ranks for comparison of external elastic membrane (EEM) and lumen inter-expert variability (IEV) between 40 and 60 MHz data. For both EEM and lumen contours at 40 and 60 MHz, the mean value and the standard-deviation (SD) are indicated. The variable *N* represents the number of contour points. Missing data correspond to contours that were not traced by at least two experts.

**Fig 3:**
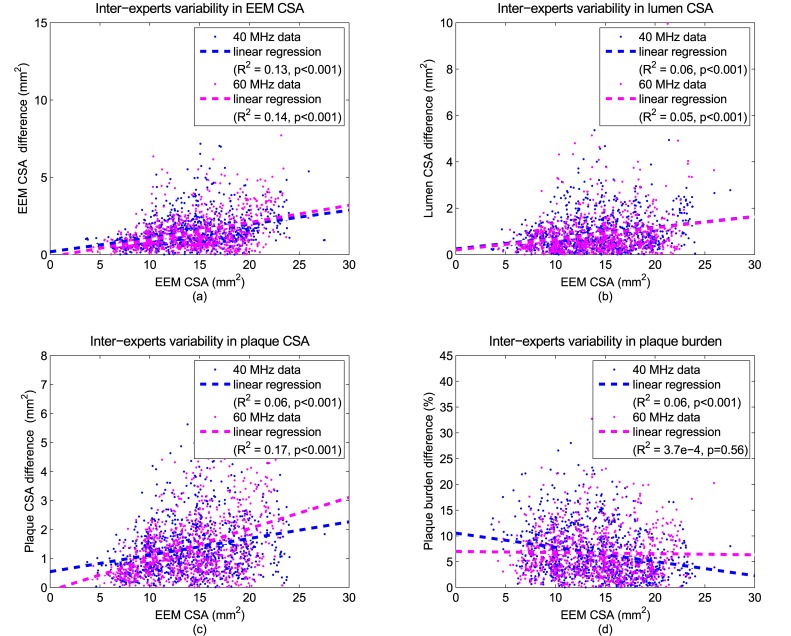
Inter-expert variability at 40 and 60 MHz. Linear regressions between inter-expert variability in external elastic membrane (EEM), lumen, and plaque cross-sectional areas (CSA), and plaque burden, on one hand, and average EEM CSA, on the other hand, at 40 and 60 MHz. Inter-expert variability in a measurement is defined as its maximal pairwise difference. The EEM CSA is defined as the average over the 3 experts’ measurement in EEM CSA obtained from their contours.

Inter-expert variability in EEM, lumen and plaque CSAs, and plaque burden were related to the average EEM CSA for both 40 and 60 MHz datasets; see Fig 3. Linear regressions indicated that differences between experts in EEM, lumen and plaque CSA measures increased with the EEM CSA (*R*^2^ ranging between 0.05 to 0.17, with *p*-values less than 0.001). At 40 MHz, the difference in plaque burden decreased with the EEM CSA (*R*^2^ = 0.06, *p* = 0.001). At 60 MHz, there was no relation between the differences in plaque burden and EEM CSA.

For 40 MHz data (Table 5), the average distances between algorithmic and experts’ EEM contours were significantly lower than IEV measures (*p* < 0.001). The average distances between algorithmic and experts’ lumen contours did not, however, differ from IEV measures (*p* = 0.623). For the 60 MHz dataset, results are summarized in Table 6. The average distances between algorithmic and experts’ EEM contours were lower than IEV measures (*p* < 0.001). The average distances between algorithmic and experts’ lumen contours were, on the other hand, higher than IEV measures (*p* < 0.001).

**Table 5:** Comparison of algorithmic to expert discrepancy with inter-expert variability at 40 MHz.

40 MHz data	EEM (*N* = 1155, 101 missing)	Lumen (*N* = 1155, 100 missing)
AED (mm)	0.113 ± 0.076	0.139 ± 0.067
IEV (mm)	0.124 ± 0.077	0.136 ± 0.080
*P*-values	*p* < 0.001	*p* = 0.623

Wilcoxon rank sum tests for comparison of external elastic membrane (EEM) and lumen algorithmic to expert discrepancy (AED) with inter-expert variability (IEV) for the 40 MHz dataset. For both EEM and lumen contours, and AED and IEV performance metrics, the mean value and the standard-deviation (SD) are indicated. The variable *N* represents the number of contour points. Missing data correspond to contours that were not traced by at least two experts.

**Table 6:** Comparison of algorithmic to expert discrepancy with inter-expert variability at 60 MHz.

60 MHz data	EEM (*N* = 1157, 227 missing)	Lumen (*N* = 1157, 216 missing)
AED (mm)	0.114 ± 0.070	0.154 ± 0.071
IEV (mm)	0.120 ± 0.079	0.129 ± 0.081
*P*-values	*p* < 0.001	*p* < 0.001

Wilcoxon rank sum tests for comparison of external elastic membrane (EEM) and lumen algorithmic to expert discrepancy (AED) with inter-expert variability (IEV) for the 60 MHz dataset. For both EEM and lumen contours, and AED and IEV performance metrics, the mean value and the standard-deviation (SD) are indicated. The variable *N* represents the number of contour points. Missing data correspond to contours that were not traced by at least two experts.

The initialisation procedure was not significantly different between 40 and 60 MHz data (*p*-value of 0.833). The time required to segment both boundaries (lumen-intima and EEM-adventitia) of each sequence was on average 8 min 18 s ± 3 min 41 s (on an Intel Core i7 1.73 GHz with 6 GB of RAM). This corresponds approximately to a processing time of 0.32 s per 2D IVUS. Optimization of the Matlab code and parallelization (on multicore CPU and on GPU) would largely improve processing time.

Finally, the average differences in EEM, lumen and plaque CSAs, and plaque burden between algorithmic and experts’ contours were lower than the inter-expert variability in these measurements at 40 MHz (*p* < 0.001); see Table 7. Similar results were observed with the 60 MHz dataset (Table 8, *p* < 0.001).

**Table 7:** Comparison of algorithmic to expert discrepancy with inter-expert variability at 40 MHz in cross-sectional areas and plaque burden.

40 MHz data	EEM CSA (mm^2^) (*N* = 1155, 101 missing)	Lumen CSA (mm^2^) (*N* = 1155, 100 missing)	Plaque CSA (mm^2^) (*N* = 1155, 101 missing)	Plaque burden (%) (*N* = 1155, 101 missing)
AED	0.896 ± 0.778	0.702 ± 0.542	0.950 ± 0.705	5.103 ± 3.598
IEV	1.435 ± 1.053	0.900 ± 0.799	1.347 ± 0.968	6.677 ± 4.866
*P*-values	*p* < 0.001	*p* < 0.001	*p* < 0.001	*p* < 0.001

Wilcoxon rank sum tests for comparison of the algorithmic to expert discrepancy (AED) in four measurements with the inter-expert variability (IEV) in these measurements on 40 MHz data: external elastic membrane (EEM) cross-sectional area (CSA), lumen, CSA, plaque CSA, and plaque burden. Mean values and standard-deviations (SD) are indicated. The variable *N* represents the number of contour points. Missing data correspond to contours that were not traced by at least two experts.

**Table 8:** Comparison of algorithmic to expert discrepancy with inter-expert variability at 60 MHz in cross-sectional areas and plaque burden.

60 MHz data	EEM CSA (mm^2^) (*N* = 1157, 227 missing)	Lumen CSA (mm^2^) (*N* = 1157, 216 missing)	Plaque CSA (mm^2^) (*N* = 1157, 227 missing)	Plaque burden (%) (*N* = 1157, 227 missing)
AED	0.934 ± 0.803	0.793 ± 0.666	1.063 ± 0.870	5.659 ± 3.790
IEV	1.405 ± 1.270	0.867 ± 0.879	1.378 ± 1.088	6.686 ± 4.821
*P*-values	*p* < 0.001	*p* < 0.001	*p* < 0.001	*p* < 0.001

Wilcoxon rank sum tests for comparison of the algorithmic to expert discrepancy (AED) in four measurements with the inter-expert variability (IEV) in these measurements on 60 MHz data: external elastic membrane (EEM) cross-sectional area (CSA), lumen CSA, plaque CSA, and plaque burden. Mean values and standard-deviations (SD) are indicated. The variable *N* represents the number of contour points. Missing data correspond to contours that were not traced by at least two experts.

## Summary and Discussion

In this work, two notions were considered: 1) the inter-expert variability between manually traced contours by 3 experts; and 2) the pointwise contour uncertainty as computed with the proposed FMM segmentation algorithm. Relations between these two notions were investigated at both 40 and 60 MHz.

### Database

Among the 2312 images selected over the 32 loops for assessment, there were *N* = 501 cross-sectional images for the right coronary artery, *N* = 725 images for the left anterior descending artery, and *N* = 1156 images for the circumflex artery. Moreover, there were *N* = 1115 images pre-intervention (547 at 40 MHz and 568 at 60 MHz) and *N* = 1197 post-intervention (608 at 40 MHz and 589 at 60 MHz). The average value ± SD of plaque burden was 53.9 ± 14.5% on the 2312 selected images.

### Local inter-expert variability and contour uncertainty

The results of Tables 2 and 3 express the overall tendency that higher values of PCU corresponded to higher values of local IEV; *i*.*e*., the contour points displayed in red with highest FMM uncertainty have a tendency to correspond to points with a higher inter-expert variability. This finding deserves attention since the two notions are conceptually distinct: the former is computed with an algorithm, whereas the latter depends on manually traced gold-standards. One application could be to rely on portions of images with low PCU to make clinical measurements (less uncertainty), since a lower inter-expert variability would be more likely to occur. Note that the cut-off value for displaying high PCU could be modified in the context of clinical scans according to user preferences, to reduce or increase the proportion of contours displayed in red (*i*.*e*., to accept more or less contour uncertainties).

### Inter-expert variability at 40 versus 60 MHz

Table 4 revealed that inter-expert variability was lower on 60 MHz than 40 MHz data for both EEM and lumen contours. Note, however, that there were approximately twice as much missing pairs of expert’ traced contours for 60 MHz versus 40 MHz data. But, when at least two experts considered that they could trace contours, the inter-expert variability was lower at 60 MHz than 40 MHz. Thus, although there was an issue that penetration depth and signal attenuation could worsen image quality at higher frequency, it was found that more reproducible manual vessel boundary delineations were obtained by experts at 60 MHz, even for EEM contours, which are located at a greater depth than luminal contours. Moreover, Fig 3 revealed that inter-expert variability in either EEM, lumen or plaque CSA increased as the EEM CSA was larger for both 40 and 60 MHz. However, the differences in plaque burden decreased at 40 MHz or remained constant at 60 MHz as EEM CSA increased. Thus, the increase in inter-expert variability with an increase of depth (corresponding to an increase in EEM CSA) presents the same tendencies at both frequencies, except for plaque burden.

### Automated segmentations within inter-expert variability

The reported tests showed that EEM, lumen and plaque CSAs, and plaque burden obtained from the algorithmic contours were within the inter-expert variability for both 40 and 60 MHz data. In [15], three other evaluation measures were proposed for assessment of IVUS segmentation algorithms: Hausdorff distance (HD) (*i*.*e*., maximal distance between two contours), percentage of area difference (PAD), and Jaccard measure (JM). For 40 MHz data, the average value of these evaluation measures between algorithmic and experts’ contours were found within inter-expert variability, except for JM on EEM contours and HD on lumen contours (results are reported in Tables 9 and 10). For 60 MHz data, the average value of these performance measures between algorithmic and experts’ contours were also within inter-expert variability, except for HD and JM on EEM contours and HD on lumen contours (results are reported in Tables 11 and 12). In particular, PAD measurements were within inter-expert variability for both 40 and 60 MHz data. Further fine tuning of the proposed segmentation method would be required to improve its performance as per average distance and HD criteria, especially for the second one, which measures the worst case scenario (*i*.*e*., the maximal distance). Nevertheless, all six area-based evaluation measures (*i*.*e*., differences in EEM, lumen or plaque CSA, plaque burden, PAD and JM) indicated that the algorithmic contours were within inter-expert variability, except for JM on EEM contours. In particular, despite the fact that inter-expert variability was lower at 60 MHz than at 40 MHz, the algorithmic contours were nevertheless within this variability. Therefore, the proposed segmentation method that was previously assessed at 20 and 40 MHz [10, 11] was found to be also within inter-expert variability at both 40 and 60 MHz in the present study.

**Table 9:** Comparison of algorithmic to expert discrepancy with inter-expert variability at 40 MHz in Balocco et al. [15] measurements (EEM).

40 MHz data	EEM HD (mm) (*N* = 1155, 101 missing)	EEM PAD (no units) (*N* = 1155, 101 missing)	EEM JM (no units) (*N* = 1155, 101 missing)
AED	0.337 ± 0.195	0.066 ± 0.053	0.815 ± 0.084
IEV	0.324 ± 0.215	0.103 ± 0.067	0.889 ± 0.060
*P*-values	*p* = 0.828	*p* < 0.001	*p* < 0.001

Wilcoxon rank sum tests for comparison of the algorithmic to expert discrepancy (AED) for external elastic membrane (EEM) contours—as measured with Hausdorff distance (HD), percentage of area difference (PAD), and Jaccard measure (JM)—with the inter-expert variability (IEV)—based on the same measures—on 40 MHz data. Mean values and standard-deviations (SD) are indicated. The variable *N* represents the number of contour points. Missing data correspond to contours that were not traced by at least two experts.

**Table 10:** Comparison of algorithmic to expert discrepancy with inter-expert variability at 40 MHz in Balocco et al. [15] measurements (lumen).

40 MHz data	Lumen HD (mm) (*N* = 1155, 100 missing)	Lumen PAD (no units) (*N* = 1155, 100 missing)	Lumen JM (no units) (*N* = 1155, 100 missing)
AED	0.392 ± 0.182	0.116 ± 0.087	0.896 ± 0.062
IEV	0.372 ± 0.236	0.145 ± 0.124	0.822 ± 0.096
*P*-values	*p* = 0.001	*p* < 0.001	*p* < 0.001

Wilcoxon rank sum tests for comparison of the algorithmic to expert discrepancy (AED) for lumen contours—as measured with Hausdorff distance (HD), percentage of area difference (PAD), and Jaccard measure (JM)—with the inter-expert variability (IEV)—based on the same measures—on 40 MHz data. Mean values and standard-deviations (SD) are indicated. The variable *N* represents the number of contour points. Missing data correspond to contours that were not traced by at least two experts.

**Table 11:** Comparison of algorithmic to expert discrepancy with inter-expert variability at 60 MHz in Balocco et al. [15] measurements (EEM).

60 MHz data	EEM HD (mm) (*N* = 1157, 227 missing)	EEM PAD (no units) (*N* = 1157, 227 missing)	EEM JM (no units) (*N* = 1157, 227 missing)
AED	0.333 ± 0.196	0.067 ± 0.050	0.798 ± 0.084
IEV	0.318 ± 0.261	0.100 ± 0.075	0.893 ± 0.061
*P*-values	*p* = 0.002	*p* < 0.001	*p* < 0.001

Wilcoxon rank sum tests for comparison of the algorithmic to expert discrepancy (AED) for external elastic membrane (EEM) contours—as measured with Hausdorff distance (HD), percentage of area difference (PAD), and Jaccard measure (JM)—with the inter-expert variability (IEV)—based on the same measures—on 60 MHz data. Mean values and standard-deviations (SD) are indicated. The variable *N* represents the number of contour points. Missing data correspond to contours that were not traced by at least two experts.

**Table 12:** Comparison of algorithmic to expert discrepancy with inter-expert variability at 60 MHz in Balocco et al. [15] measurements (lumen).

60 MHz data	Lumen HD (mm) (*N* = 1155, 216 missing)	Lumen PAD (no units) (*N* = 1155, 216 missing)	Lumen JM (no units) (*N* = 1155, 216 missing)
AED	0.435 ± 0.199	0.124 ± 0.092	0.893 ± 0.061
IEV	0.367± 0.267	0.136 ± 0.127	0.834 ± 0.093
*P*-values	*p* < 0.001	*p* < 0.001	*p* < 0.001

Wilcoxon rank sum tests for comparison of the algorithmic to expert discrepancy (AED) for lumen contours—as measured with Hausdorff distance (HD), percentage of area difference (PAD), and Jaccard measure (JM)—with the inter-expert variability (IEV)—based on the same measures—on 60 MHz data. Mean values and standard-deviations (SD) are indicated. The variable *N* represents the number of contour points. Missing data correspond to contours that were not traced by at least two experts.

Note that the IEV measurements were based on radial scanlines, which pass through the center of the catheter. Another measurement would be based on lines passing through the center of the artery. This latter measurement, which is based on an anatomical criterion, could be used as an improvement of the IEV concept in future work.

### Comparison with previous studies

For comparison with results reported in the literature, the interobserver intraclass correlation coefficients (ICCs) were computed for EEM, lumen and plaque CSAs for 40 and 60 MHz data with the statistical software R (ICC with 3 raters). The interobserver ICCs at 40 MHz were 0.98, 0.95 and 0.95 for EEM, lumen and plaque CSAs. At 60 MHz, the interobserver ICCs for these measures were 0.98, 0.94 and 0.95. These values are comparable to the interobserver ICCs reported in the literature. In [16], interobserver ICCs (with two observers) were respectively 0.98, 0.95 and 0.95 for EEM, lumen and plaque volumes on 30 MHz data. In [17], interobserver ICCs (with 3 observers) were 0.988 and 0.991 for EEM and lumen CSAs, respectively, also on 30 MHz data.

To compare discrepancy between algorithmic and experts’ contours with a previous standardization and evaluation study [15], the mean pairwise HD between algorithmic and experts’ EEM and lumen contours were 0.337 and 0.392 mm, respectively, as reported in Tables 9 and 10. In Table 5 of [15], the best of these measures over 8 independent participants of this evaluation study were higher at 1.15 and 1.16 mm, respectively. The average algorithmic to experts’ discrepancy as measured with PAD was 0.066 and 0.11 for EEM and lumen contours, respectively (c.f. Tables 9 and 10), whereas in Table 5 of [15], the best values were comparable to our results at 0.10 for both EEM and lumen contours. Lastly, in the present study, JM measures between algorithmic and experts’ contours were 0.815 and 0.896 for EEM and lumen contours, respectively. In [15], the best JM measures were 0.85 and 0.86, respectively.

### Artefacts due to movements of the catheter

Contraction of the myocardium and movements of the heart occurring during the cardiac cycle may cause longitudinal displacements of the catheter up to 6.5 mm [18]. However, these movements of the catheter are identical whether images are acquired at 40 or 60 MHz (because only the center frequency of the excitation was changed, not the type of catheter or transducer), or whether contours are manually delineated or algorithmically computed (because these are post-processing steps). Thus, variability of contours during the cardiac cycle due to heart movements was not a confounding variable in our analysis. Moreover, ECG-gated data, which is desirable for the purpose of volumetric measurements, is expected to present no further difficulties for the proposed segmentation algorithm, since the harder case of non ECG-gated data has been assessed successfully in the present study.

## Conclusions

Display of FMM contour uncertainty may guide clinicians in choosing reliable images or portions of images for quantitative measurements. Inter-expert variability was significantly lower on 60 MHz data than on 40 MHz data, although there were twice as many contours that could not be traced by at least two out of three experts on 60 MHz data. These findings indicate that IVUS of coronary arteries might be preferably imaged at 60 MHz than at 40 MHz. Differences in EEM, lumen and plaque cross-sectional areas, and plaque burden based on algorithmic contours were within inter-expert variability in these measurements for both 40 MHz and 60 MHz data. Thus, the proposed segmentation algorithm yields, at both frequencies, measurements with a reliability comparable to that of measurements based on experts’ manual delineation of EEM and lumen contours.

## Appendix A: Segmentation Method

The implementation of the FMM segmentation algorithm used in the reported tests follows that of [11], except for the following adaptations. The main modifications are as follows:

1. In the EEM region-based speed function, the PDFs for the B-mode values conditional to 4 underlying tissues, which are used to compute posterior probabilities, are now described with a non-parametric model (based on histograms) rather than a mixture of gamma distributions model. The rationale for this modification is that the estimation procedure for the non-parametric model was faster than the one for the parametric mixture model, while segmentation results were very similar.
2. In the lumen region-based speed function, the posterior probabilities that are used to define the EEM region-based speed function are now used to classify pixels into 4 labels according to the type of underlying tissues. These labels are then used to define the lumen region-based speed function. Whereas the presence of a contrast between the PDF of the intima-media layers and the one of the adventitia allows distinguishing between these two types of tissues using posterior probabilities, it was found more convenient to use labels to distinguish between lumen pixels and intima pixels.
3. In the contour-based term of the EEM speed function, a gamma correction function implemented as a lookup table is applied to the response of the image to a gradient filter to exploit gradient directivity.
4. In the contour-based term of the lumen speed function, a Gabor filter is applied to the B-mode image rather than a gradient filter. A gamma correction function is also applied to the image response. Whereas a gradient filter for the EEM can detect the contrast in echogenicity due to the media, a Gabor filter was chosen to detect a difference in texture between the lumen and the intima layer.”

### EEM region-based speed function

The first modification concerns the posterior probabilities *P*(*s* ∈ *m* | *A*_*s*_) and *P*(*s* ∈ *n* | *A*_*s*_) appearing in Eq. (1) of [11], which describes the texture gradient intervening in the EEM region-based speed function. Here, *s* represents a pixel of the image, *m* and *n* are regions comprising the intima and lumen, respectively, and *A*_*s*_ is the gray level of pixel *s*. These posterior probabilities are computed from likelihoods *P*(*A*_*s*_ | *m*) and *P*(*A*_*s*_ | *n*), which were previously estimated as mixtures of gamma probability distributions. As it turned out, the segmentation results based on this mixture model were very close to the ones obtained with a non-parametric model. Since, the computation of non-parametric histograms is more rapid than the Expectation-Maximization (EM) algorithm [19] used for the parametric model estimations, the following non-parametric model was chosen.

As mentioned in Section 2.2 of [11], four classes of tissues/artifacts were considered for the EEM and lumen segmentation corresponding to four regions: 1) a region comprising the guide wire and the lumen (class *c* = 1); 2) a region comprising the intima (inside the intima-media) (class *c* = 2); 3) a region comprising the media (inside the EEM) (class *c* = 3); and 4) the surrounding tissues (outside the EEM) (class *c* = 4), respectively, as well as any other artefacts present within the tissue (calcium, its shadow cone, stent, side branches, etc …). On each class *c*, a non-parametric probability density function (PDF) model for the gray level amplitudes, in the form of a normalized histogram, was adopted. The PDFs *P*(*A*_*s*_ | *c*) for tissues *c* = 1, 2, 3 and 4 were estimated based on the initial interpolated EEM and lumen boundaries discussed in Section 2.4 of [11]; *i*.*e*., the blue contours in the right panel of Fig. 3 in [11]. Moreover, in order to avoid empty bins in the resulting PDFs—to prevent division by 0 in Eq. (1) of [11]—, the non-parametric PDF was mixed with a uniform distribution on bins (with constant probability 1/256) with a small weight of 10^−6^ in the mixture.

### Lumen region-based speed function

The second modification concerns the lumen region-based speed function. Each pixel was assigned the unique tissue label *c* for which the posterior distribution *P*(*s* ∈ *c* | *A*_*s*_) ∝ *P*(*A*_*s*_ | *c*) was maximal. The proportions of pixels in the neighborhoods *v* and *w* of the propagating boundary (see Fig. 2 in [11]) that are labeled by class *c* are denoted *P*_*c*_ and *Q*_*c*_, respectively. The following speed functions were then considered in the new implementation, where *μ* is a constant:

$$F_{m,n,Labels}(i,j)={\left(1+(P_{m}+1-Q_{m}-\sum_{c=1}^{4}P_{c}Q_{c})/\mu \right)}^{-1},$$ (A-1)

if *m* represents the lumen and *n* the intima, or

$$F_{m,n,Labels}(i,j)={\left(1+(1-\sum_{c=1}^{4}P_{c}Q_{c})/\mu \right)}^{-1},$$ (A-2)

if *m* represents the intima and *n* the lumen.

Note that in Eq (A-1), the corresponding label gradient $\left(P_{m}+1-Q_{m}-\sum_{c=1}^{4}P_{c}Q_{c}\right)$ is maximal if all pixels inside *v* are labeled as belonging to tissue class *m* (*i*.*e*., the lumen), none of the pixels inside *w* are labeled as belonging to *m*, and the class labels appearing in one neighborhood are distinct from the ones appearing in the other neighborhood. In Eq (A-2), the corresponding label gradient $\left(1-\sum_{c=1}^{4}P_{c}Q_{c}\right)$ is maximal if all class labels appearing in one neighborhood are distinct from the ones appearing in the other neighborhood.

### EEM contour-based speed function

The last modifications described here concern the EEM and lumen contour-based speed functions of Eq. (2) in [11]. For the EEM interface, the contour-based speed function now uses a gray level gradient that exploits the echogenicity directivity. Namely, the passage from the media to the adventitia usually consists in an increase of echogenicity. For this purpose, high values of the gray level gradient were considered. For the lumen interface, the gray level gradient was replaced with a textural filter, the Gabor filter [20], which takes into account the speckle present in the lumen, especially at high frequency.

**Fig 4:**
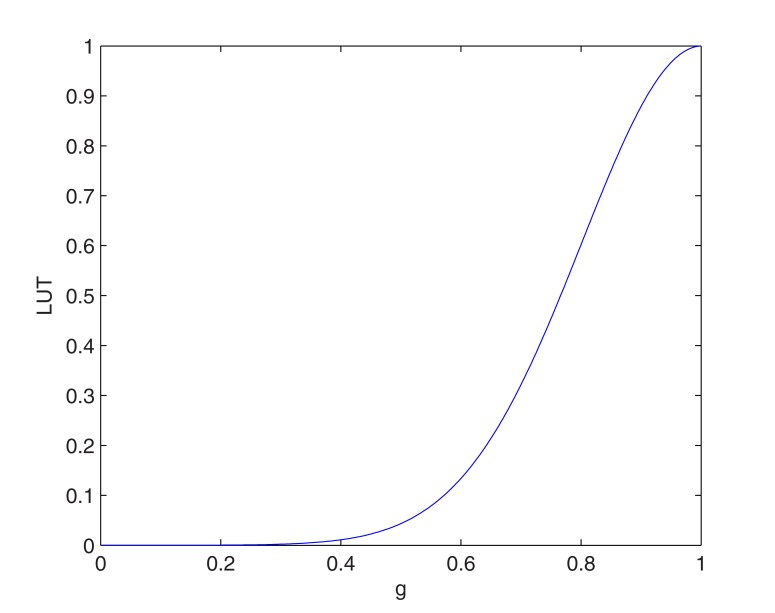
Gamma correction function implemented as a lookup table (*LUT*). Illustration of the *LUT* function of Eq (A-4) used in Eqs (A-3) and (A-5).

In details, for the EEM interface, the contour-based component is now of the form:

$$F_{dir.grad}(i,j)=(1+M_{3\times 3}*LUT{\left(\nabla M_{15\times 5}*A_{s})/\lambda \right)}^{-1},$$ (A-3)

where *M*_3×3_ is a 3 × 3 (pixels) mean filter, *LUT* denotes a gamma correction function implemented as a lookup table, and where ∇ is the gradient operator corresponding to the filter [-1; 00…00 (14 zeros);1] in the radial direction, and *M*_15×5_ is a 15 × 5 (pixels) mean filter (playing the role of a low-pass filter). In this equation, *λ* is a normalizing constant that was calibrated with the constant *μ*, as described in Section 2.2 of [11]. The function *LUT* is illustrated in Fig 4 and its analytical expression is of the form:

$$LUT(g)=e^{-\frac{g^{2}}{2\alpha^{2}}},$$ (A-4)

where *g* is the normalized filter response (*i*.*e*., lying between 0 and 1, based on the minimal and maximal values of the filter response), and *α* = 0.2 is the standard-deviation of this Gaussian function. The purpose of this filter was to favor large values of increase in echogenicity, thus corresponding to positive values of the normalized filter response, and hence of the gray level gradient.

### Lumen contour-based speed function

For the lumen interface, the contour-based component is now of the form:

$$F_{dir.\;Gabor\;filt.}(i,j)=(1+M_{3\times 3}*LUT{\left(GF*A_{s})/\lambda \right)}^{-1},$$ (A-5)

where *M*_3×3_ is a 3 × 3 (pixels) mean filter, *GF* denotes a 23 × 23 pixels Gabor filter with radial frequency shift *μ*_*x*_ (see Eq (A-6)) and standard deviation $\tilde{\sigma }=\frac{\left(2^{\text{b}}-1\right)}{\left(2^{\text{b}}+1\right)}\mu_{\text{x}}\frac{1}{\sqrt{2\text{log}2}}$, corresponding to one octave (*i*.*e*., *b* = 1) [21]. Namely, the function *GF* is defined by the expression:

$$GF(x,y)=e^{-2\pi^{2}{\tilde{\sigma }}^{2}(x^{2}+y^{2})}\text{sin}(2\pi \mu_{x}x).$$ (A-6)

**Fig 5:**
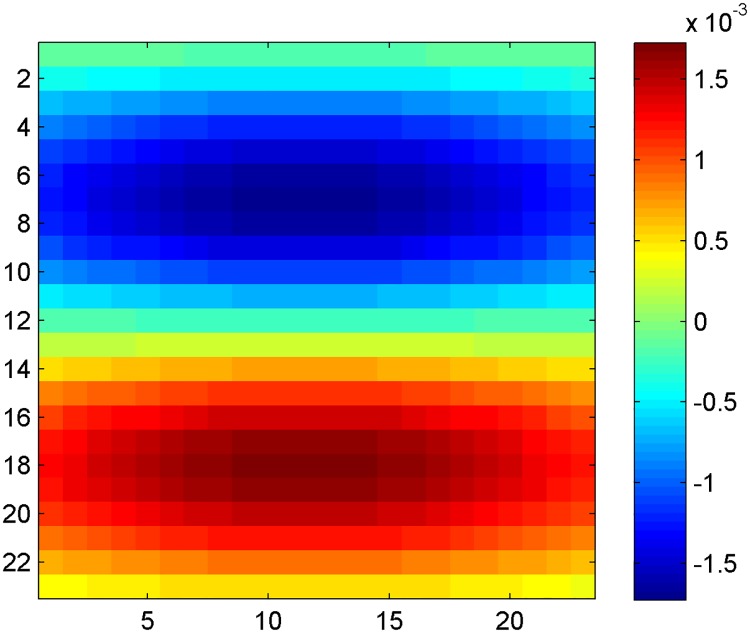
Gabor filter. Illustration of the 23 × 23 pixels Gabor filter of Eq (A-6).

See Fig 5 for an illustration of this filter. The parameter $\tilde{\sigma }$ corresponds to the standard deviation of a (shifted) Gaussian filter in the frequency domain. The corresponding modulated Gaussian filter in the spatial domain (*i*.*e*., the Gabor filter) has a standard deviation equal to $\sigma =\frac{1}{2\pi \tilde{\sigma }}\approx 13.5$ pixels (from which the values of *μ*_*x*_ and $\tilde{\sigma }$ can be deduced). As for the gradient filter, the look-up table function *LUT* of Eq (A-4) was used to enhance positive values of the Gabor filter response.

Globally, the proposed speed function of the current study was selected as the average of the components given in Eq. (1) of [11] and Eq (A-3) for the EEM, or Eqs (A-1) or (A-2) and (A-5) for the lumen, respectively:

$$F_{EEM}(i,j)=\frac{1}{2}F_{m,n,PDF}(i,j)+\frac{1}{2}F_{dir.\;grad}(i,j);$$ (A-7)

$$F_{lumen}(i,j)=\frac{1}{2}F_{m,n,Labels}(i,j)+\frac{1}{2}F_{dir.\;Gaborfilt.}(i,j).$$ (A-8)

Since the adopted values of the constants *μ* (in Eq. (1) of [11], and Eqs (A-1) and (A-2)) and *λ* (in Eqs (A-3) and (A-5)) were estimated independently for the EEM and lumen boundaries, they allowed to calibrate the contribution of each type of gradient in the combined speed function.

**Fig 6:**
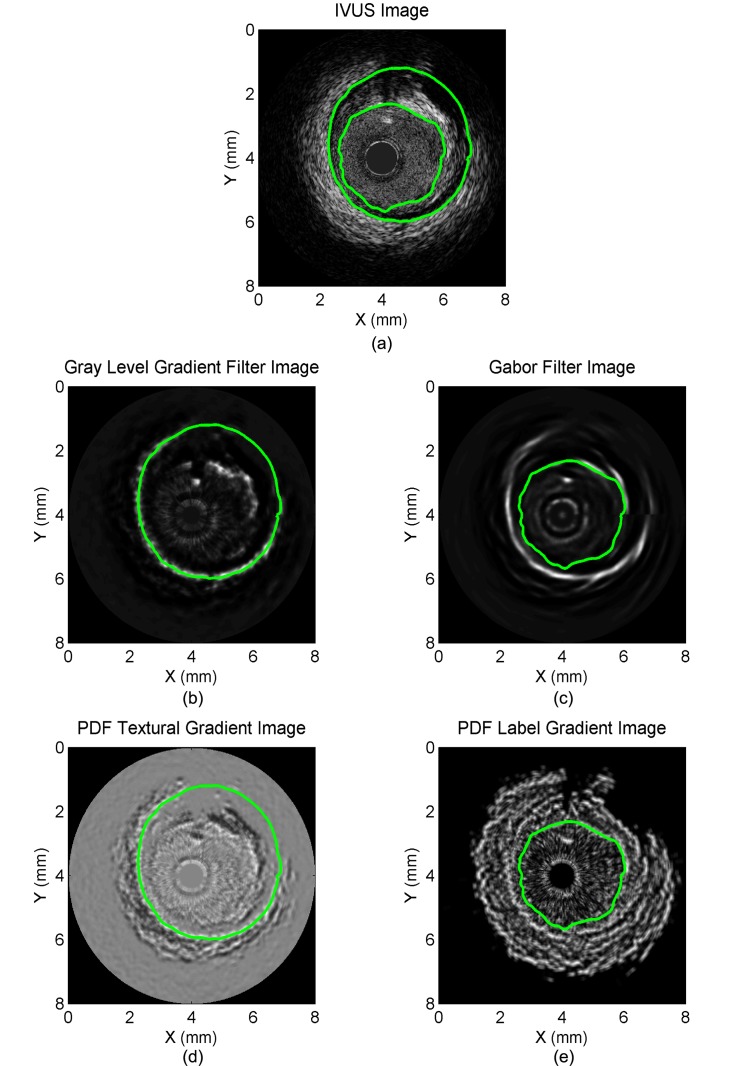
Example of an IVUS image and corresponding gradient images. Panel a) displays an IVUS image. Panels b) and d) display gradient images obtained by processing the image with a gray level gradient filter (panel b)) and with a probability density function (PDF) textural gradient filter (panel d)), respectively. The former gradient filter reveals contour-based information, whereas the latter filter detects region-based information. Both types of information are combined into the speed function of the fast-marching method (FMM) algorithm used in the external elastic membrane (EEM) contour segmentation (c.f. Eq (A-7)). The resulting EEM contour is superimposed on both gradient images (green full curve). Panels c) and e) display gradient images obtained by processing the image with a Gabor filter (panel c)) and with a PDF label gradient filter (panel e)), respectively. As for the EEM, the former gradient filter reveals contour-based information, whereas the latter detects region-based information. Both types of information are combined into the speed function of the FMM algorithm used in the lumen contour segmentation (c.f. Eq (A-8)).

To illustrate the gradients used in the computation of the speed functions of Eq. (1) of [11], and Eqs (A-1), (A-3) and (A-5), parametric images are displayed in Fig 6.

## Appendix B: Semi-Automatic Initialization of the FMM Segmentation Algorithm

**Fig 7:**
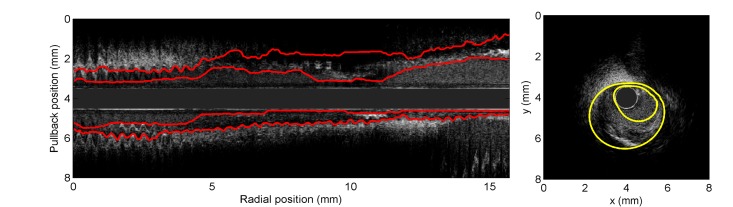
Semi-automatic initialization. Left: example of initial semi-automatically traced contours (red curves) on a longitudinal cut of a pullback. Right: example of a cross-section from the same pullback; the yellow curves represent the contours obtained by a B-spline interpolation of the points obtained from the manually traced boundaries on the L-views. The initial boundaries used by the algorithmic segmentation are obtained from these yellow contours.

As mentioned above, the implementation of the FMM requires initial contours representing approximate boundaries between the lumen and the intima, and between the media and the adventitia, respectively. The initial radial contours of all frames in both implementations were determined from semi-automatically traced boundaries on two longitudinal cuts (L-views) of the IVUS sequence corresponding to two planes at equally spaced angles (see the left panel of Fig 7). By considering the 2 L-views, this yielded four contour points in each 2D frame that were spline-interpolated radially to obtain each closed approximate initial boundary (lumen-intima and media-adventitia) that were forbidden to overlap (see Fig 7, right panel).

The longitudinal lines were produced semi-automatically with dynamic programming (see Section 8.2 in [13]) using information provided by points on L-views. In particular, produced longitudinal lines followed more precisely lumen-intima or media-adventitia boundaries.

The cost function that was minimized with the implemented dynamic programming algorithm is of the form:

$$\begin{array}{l} {\text{Cost}}_{Total}(path)=w_{region}\times \sum_{n=1}^{N}{\text{Cost}}_{region}(path(n))+w_{contour}\times \sum_{n=1}^{N}{\text{Cost}}_{contour}(path(n))\\ \;+w_{smooth}\times \sum_{n=2}^{N}dist^{2}(path(n-1),path(n))\\ \;+w_{user}\times \sum_{n=2}^{N}dist^{2}(path(n-1),userpath(n)) \end{array}$$ (B-1)

where *N* is the total number of points in the path (*i*.*e*., the number of frames in the IVUS sequence), *w*_*region*_, *w*_*contour*_, *w*_*smooth*_, *w*_*user*_ are non-negative weights, *Cost*_*region*_, *Cost*_*contour*_ are cost functions described below, and *dist* denotes the Euclidean 2D-distance.

The first term, a region-based cost, used a label map (based on gamma distributions) of the L-view (explained below). The second term, a contour-based term, corresponds to the contour-based speed functions of the FMM. The third term, a smoothing term, was added in the cost function to prevent prohibitive large distances between consecutive points in the contours. The fourth term encourages a path close to the initial points in L-views (*userpath*).

For the EEM contours, the weights appearing in Eq (B-1) were set empirically to *w*_*region*_ = 0.5, *w*_*contour*_ = 1, *w*_*smooth*_ = 0.02, *w*_*user*_ = 0. For the lumen contours, it was found convenient to take the weights *w*_*region*_ = 0.5, *w*_*contour*_ = 1, *w*_*smooth*_ = 0.1, *w*_*user*_ = 0.0008. Lastly, a prior on the initial points in L-views was considered for lumen contours (not for EEM contours). We privileged a higher smoothing constraint for the lumen than for the EEM contours, because lumen contours in the tested images were usually less precisely defined than EEM contours. For the same reason, it was preferable to favor user input in the case of lumen contours.

For the region-based cost function, the EM algorithm [19, 22] was used to estimate a mixture of 5 gamma distributions,

$$P(A_{s})=\sum_{l=1}^{5}p_{l}G(A_{s}|k_{l},\theta_{l}),$$ (B-2)

for the gray levels in the L-view. See Eq. (4) in [11] for a definition of the gamma distribution. The number of gamma distributions was chosen so as to allow fitting the mixture to the histogram of gray levels in the L-view. Thus, there was for each label *l* = 1,…, *l* = 5, a gamma distribution $G(A_{s}|k_{l},\theta_{l})$. Then, for each pixel *s* in the L-view, one could determine (in the sense of the maximum likelihood), the unique gamma label *l* for which $\text{log}G(A_{s}|k_{l},\theta_{l})$ had the highest value. In our implementation, the log likelihoods were actually averaged over a window of size 5 × 5 pixels to obtain a spatially smoother labeling of pixels. A cost function was then computed as follows:

$${\text{Cost}}_{region}(path(n))=-0.5\times \sum_{l=1}^{5}P_{l}(1-Q_{l})+Q_{l}(1-P_{l})=\sum_{l=1}^{5}P_{l}Q_{l}-1,$$ (B-3)

where the index *l* = 1, 2, 3, 4, 5 represents each gamma label; *P*_*l*_ and *Q*_*l*_ are the proportions of pixels with label *l* in windows of size 7 × 5 on each side of the point *path*(*n*) (respectively toward the catheter and toward the outer image in the radial direction; and both centered longitudinally on the position of *path*(*n*)).

The contour-based cost function of the EEM was defined as

$${\text{Cost}}_{contour}(path(n))=-LUT(\nabla M_{15\times 5}*A_{s}),$$ (B-4)

where *A*_*s*_ is the gray level at the pixel *path(n)*, similarly to the contour-based speed function of the FMM of Eq (A-3). For the lumen, the contour-based cost function was expressed as

$${\text{Cost}}_{contour}(path(n))=-LUT(GF*A_{s}).$$ (B-5)
